# A preliminary investigation into the impact of shock wave therapy and sonotherapy on postural control of stepping tasks in patients with Achilles tendinopathy

**DOI:** 10.3389/fneur.2023.1157335

**Published:** 2023-06-02

**Authors:** Magdalena Stania, Michał Pawłowski, Wojciech Marszałek, Grzegorz Juras, Kajetan Jacek Słomka, Piotr Król

**Affiliations:** Institute of Sport Sciences, Academy of Physical Education, Katowice, Poland

**Keywords:** Achilles tendon—injuries, postural control (MeSH), extracorporeal shockwave therapy (ESWT), ultrasonic waves, therapeutics

## Abstract

**Objective:**

The outcomes of physical therapy are commonly assessed with subjective scales and questionnaires. Hence, a continuous search to identify diagnostic tests that would facilitate objective assessment of symptom reduction in those patients with Achilles tendinopathy who undergo mechanotherapy. The main aim of this study was to evaluate and compare the effectiveness of shock wave and ultrasound treatments, using objective posturographic assessment during step-up and step-down initiation.

**Materials and methods:**

The patients with non-insertional Achilles tendinopathy and pain lasting for more than 3 months were randomly assigned to one of the experimental groups, i.e., radial shock wave therapy (RSWT), ultrasound therapy, or placebo ultrasound. All groups also received deep friction massage as the primary therapy. The transitional locomotor task was performed with the affected and unaffected limb in random order, on two force platforms under two conditions (step-up and step-down). The recording of center of foot pressure displacements was divided into three phases: quiet standing before step-up/step-down, transit, and quiet standing until measurement completion. Pre-intervention measurements were performed and then short-term follow-ups at weeks 1 and 6 post-therapy.

**Results:**

The three-way repeated measures ANOVA showed few statistically significant two-factor interactions between therapy type, time point of measurement and the type of the locomotor task. Significant increases in postural sway were observed in the entire study population throughout the follow-up period. Three-way ANOVAs revealed a group effect (shock wave vs. ultrasound) on almost all variables of the quiet standing phase prior to step-up/step-down initiation. Overall, postural stability before the step-up and step-down tasks appeared to be more efficient in patients who had undergone RSWT compared to the ultrasound group.

**Conclusion:**

Objective posturographic assessment during step-up and step-down initiation did not demonstrate therapeutic superiority of any of the three therapeutic interventions used in patients with non-insertional Achilles tendinopathy.

**Clinical Trial Registration:** The trial was prospectively registered in the Australian and New Zealand Clinical Trials Registry (no. ACTRN12617000860369; registration date: 9.06.2017).

## Introduction

Achilles tendinopathy is a common term for chronic Achilles tendon pain and impaired function caused by chronic stress and overuse (1). The condition results from a failed healing response to overuse lesions in the myotendinous unit (2). Repeated overloading and microtrauma to the tendon initially lead to reactive tendinopathy, which may progress over time to degenerative tendinopathy (3, 4) with characteristic disruption of collagen fibre structure, tenocyte proliferation and increased synthesis of non-collagenous matrix (2).

Achilles tendinopathy predominantly affects athletes. According to the most recent data (5), the overall prevalence of Achilles tendinopathy in physical exercise is 6%, with the highest prevalence among gymnasts and ball games players. A cross-sectional study of Albers et al. (6) revealed the prevalence rate of Achilles tendinopathy was also high in non-athletic population (25 per 1,000 person-years). The prevalence of Achilles tendinopathy increases with age, reaching 8% in people over 45 years of life (5).

Patients with Achilles tendinopathy typically present with localized pain and swelling within the tendon aggravated by tendon loading activities (7) and prolonged rest (8). The condition significantly reduces the patient’s health-related quality of life (9, 10), and lowers their socioeconomic status due to reduction in work productivity (10).

A large variety of conservative treatments are used in the initial stages of Achilles tendinopathy including physicalmodalities, prolotherapy, *platelet-rich plasma,* corticosteroid *injections* (7), and *exercise rehabilitation* (11, 12). Shock wave and ultrasound wave use mechanical stimuli (with entirely different physical characteristics) to induce mechanotransduction that initiates repair and remodelling processes in damaged tissues (13–16)^.^ To date, the therapeutic efficacy of these two methods has been primarily assessed on the basis of the patient’s subjective report (17–23).

Degenerative changes to the Achilles tendon alter its mechanical properties (24, 25) with resultant changes in ankle biomechanics and lower limb dynamics during gait (26, 27). These abnormalities in gait biomechanics can occur during stair ascent and descent, which are everyday activities and can result in pain, injury, and decreased mobility; therefore, a proper understanding of gait biomechanics is essential for rehabilitation and injury prevention. The gait pattern for ascending and descending stairs is distinct from that of normal walking and running. The gait pattern during stair ascent and descent necessitates the coordination of multiple joints and muscles in the lower extremities to maintain balance and stability, as well as to generate the force required to overcome gravity (28). The gait cycle is adjusted while stair negotiating to account for the change in elevation and the altered demands placed on the body. During stair ascent and descent, ankle plantar flexors must oppose the external ankle dorsiflexion moment (29). In a study of (30), patients with Achilles tendinopathy used lower peak ankle plantar flexor power during stair ascent, and thus a lower concentric plantar flexor output, compared to healthy controls. The authors hypothesized this strategy might have been used to decrease loading on the Achilles tendon and reduce symptoms.

Our research hypothesis was that reduction in Achilles tendinopathy symptoms after shock wave and ultrasound therapy (19, 31) would alter the dynamics of the step-up and step-down initiation tasks. It was also assumed that the magnitude of changes in dynamic locomotor tasks observed after a series of treatments would depend on therapy type, time elapsed after therapy completion, and the difficulty of the locomotor task. Since the mechanical impact of the shock wave on the tissues is greater than that of the ultrasound wave (13, 16), we would expect greater differences in postural sway in patients undergoing shock wave therapy compared to patients on ultrasound intervention. Furthermore, considering the fact of stair ascent being a more demanding biomechanical task than stair descent (29), we formulated a research hypothesis that our patients would have more difficulty controlling postural balance on step-up compared to step-down initiation.

The main objective of this randomized controlled study was to evaluate the effectiveness of shock wave and ultrasound therapy in step-up and step-down tasks in patients with non-insertional Achilles tendinopathy. It was hoped that the obtained results would support the use of posturographic testing in comprehensive diagnostic assessment of patients with Achilles tendinopathy.

## Materials and methods

Based on the Consolidated Standards of Reporting Trials (CONSORT) guidelines (32), this is a randomized controlled trial, comparing three experimental groups, i.e., radial shock wave therapy (RSWT) (group A), ultrasound therapy (US) (group B), and placebo ultrasound (P-US) (group C). The study protocol was approved by the local Research Ethics Committee (approval number: 5/2016). The entire research project “Objective and subjective assessment of the efficacy of radial shock wave therapy and sonotherapy in Achilles tendinopathy” was prospectively registered in the Australian and New Zealand Clinical Trials Registry (no. ACTRN12617000860369; registration date: 9.06.2017). This publication presents part of the project.

The experiment was conducted in the physiotherapy outpatient unit and the Human Motor Behavior laboratory of the Academy of Physical Education in Katowice, Poland, from October 2017 through May 2022. All patients provided informed written consent to participate. This randomized controlled study followed the recommended standards for reporting participant characteristics in tendinopathy research (33).

### Patients

Patients currently seeking care and referred to outpatient orthopedic and physiotherapy units with symptoms of non-insertional Achilles tendinopathy were assessed by a clinician based on medical history and physical examination (including ultrasound scan). The clinician was blinded and not aware of the study details. The following criteria of inclusion were used (31): (1) pain over the main body of the Achilles tendon 2–6 cm proximal to its insertion; (2) pain present for more than 3 months; (3) midportion tendon abnormalities identified on ultrasound; (4) recreationally active patients, who participated in moderate-intensity aerobic activity for a total of 80 min once or twice a week (34). The exclusion criteria had been described in detail in our first report on the above mentioned project (31). To assess the baseline severity of symptoms and disability we used the activity-related pain intensity ratings on a visual analogue scale (VAS) and the Victorian Institute of Sport Assessment-Achilles (VISA-A) score, respectively (Table 1). No loading tests were used. *Pain on activity* and *disability* are among the nine core health-related domains for tendinopathy that should be used when reporting outcomes in clinical trials (35).

**Table 1:** Characteristics of study participants.

Variable	Group A (*n* = 13)		Group B (*n* = 13)		Group C (*n* = 13)		*p*-value
	$\bar{x}$	SD	$\bar{x}$	SD	$\bar{x}$	SD	
Age (year)	42	11.42	36.69	11.57	34	11.32	*p* > 0.05*
Sex	F – *n* = 2; 15.4%***		F – *n* = 4; 30.8%		F – *n* = 8; 61.5%***		0.04**
	M – *n* = 11; 84.6%		*M* – *n* = 9; 69.2%		M – *n* = 5; 38.5%		
BMI	BMI <25 kg/m² – *n* = 9; 69.2%		BMI <25 kg/m² – *n* = 6; 46.2%		BMI <25 kg/m² – *n* = 9; 69.2%		*p* > 0.05**
	BMI 25–29.9 kg/m² – *n* = 3; 23.1%		BMI 25–29.9 kg/m² – *n* = 6; 46.2%		BMI 25–29.9 kg/m² – *n* = 2; 15.4%		
	BMI >30 kg/m² – *n* = 1; 7.7%		BMI >30 kg/m² – *n* = 1; 7.7%		BMI >30 kg/m² – *n* = 2; 15.4%		
Measured body weight (kg)	80.46 ± 10.95		79.46 ± 9.25		73.53 ± 16.08		*p* > 0.05*
Measured standing height (m)	1.79 ± 0.09		1.79 ± 0.08		1.77 ± 0.12		*p* > 0.05*
Location of symptoms	Left limb – *n* = 6; 46.2%		Left limb – *n* = 4; 30.8%		Left limb – *n* = 4; 30.8%		*p* > 0.05**
	Right limb – *n* = 7; 53.8%		Right limb – *n* = 9; 69.2%		Right limb – *n* = 9; 69.2%		
Duration of symptoms (mo)	8.84	8.68	9.07	7.64	7.53	5.07	*p* > 0.05*
Activity-related pain (VAS)	6	1.9	5	2.2	5.46	2.26	*p* > 0.05*
VISA-A score	65.54	20.06	74.23	12.98	72.85	12.37	*p* > 0.05*

**Figure 1:**
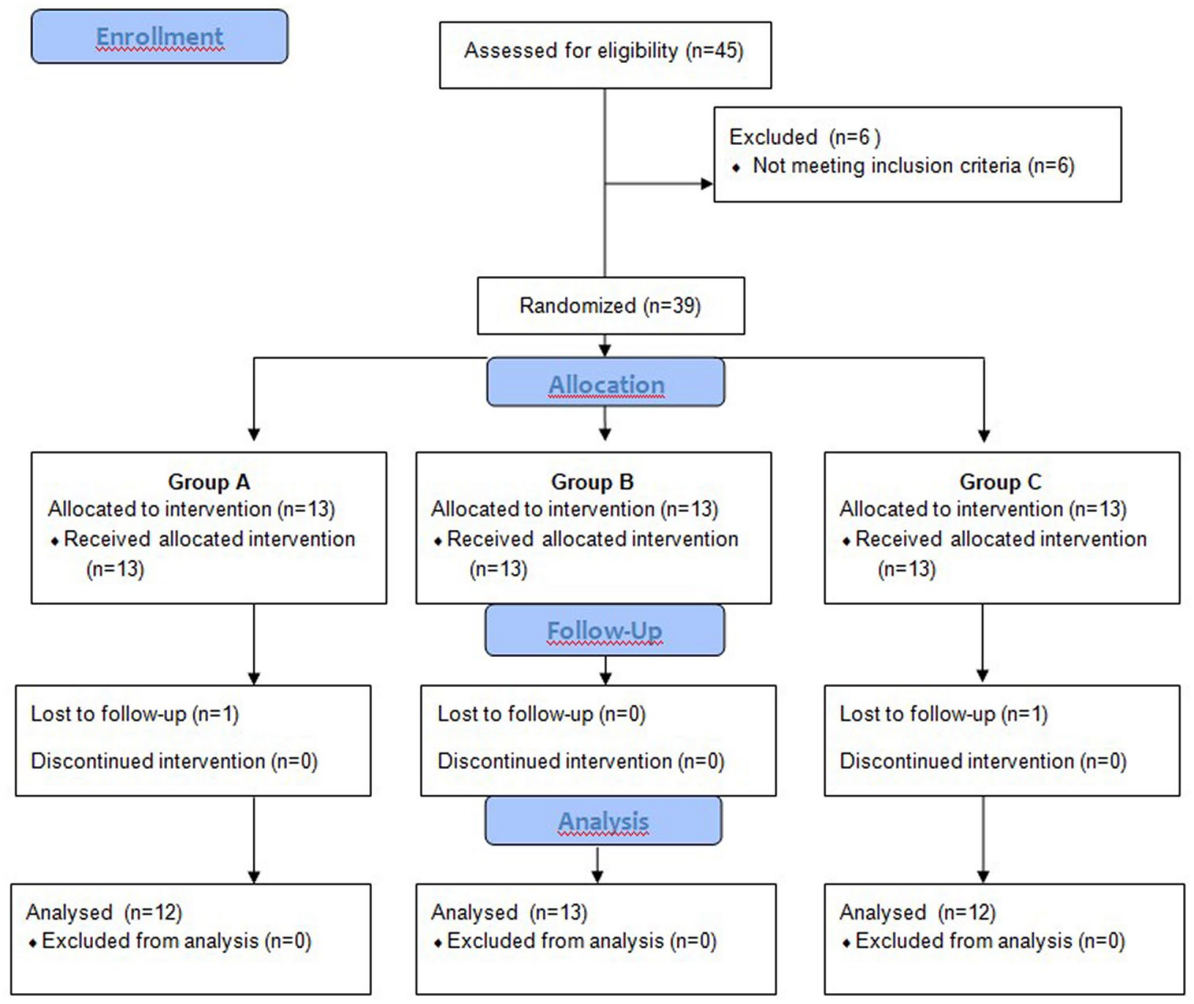
Flowchart of the trial from the baseline.

Initially, there were 45 patients with non-insertional Achilles tendinopathy, six of whom were excluded as they did not meet the inclusion criteria (Figure 1). They were all in good general condition and did not require emergency medical treatment for any other reason. In return for participating in the research project, all patients received free therapy; no payment was offered.

The patients were randomly allocated to one of the three experimental groups using *sealed opaque numbered envelopes*: group A: RSWT, group B: US, group C: P-US.

### Therapy

The following mechanotherapy parameters were applied in groups A and B:

RSWT (group A): 3 bars/10 Hz/2,000 shocks applied to the Achilles tendon, another 2,000 shocks applied to the gastrocnemius muscle/1 session a week for 3 weeks.

Sonotherapy (group B): 3 MHz/1.0 W/cm^2^/50%/each square centimeter was exposed to ultrasonic energy for 2 min/1 session a day for 10 (2 weeks).

In group C (placebo sonotherapy), all ultrasound device parameters and therapy procedures were identical as in group B except that the transducer did not generate sound waves (31).

Group B and C patients were blinded to the type of ultrasound therapy assignment (real or placebo ultrasound). However, since RSWT receivers tend to report transient but noticeable side effects, we believed group A patients could not be blinded to the treatment and no placebo group was formed.

According to the experimental design, the patients agreed not to use any other form of tendinopathy treatment when participating in the research, but were allowed to take paracetamol in a daily dose of up to 4,000 mg (36). However, since it would have been unethical to leave patients in the placebo group without any therapy for such a long period, deep friction massage was used in all study groups during the first 2 weeks of the experiment.

### Outcome assessment

Before the intervention (baseline) and at weeks 1 and 6 post-therapy, a posturography test was performed during step-up and step-down tasks. All assessments were carried out by one investigator, who had been blinded to treatment group allocation. Two patients did not complete the study and dropped out without the posturographic measurements at 6 weeks post-therapy (Figure 1).

Dynamic posturography was carried out using two (A and B) force platforms (AMTI, AccuGait, Watertown, MA, United States). The COP signals transmitted from the platforms were amplified and sampled at a frequency of 100 Hz with the AMTI NetForce software and then filtered at 6 Hz using dual-pass Butterworth digital filter with MATLAB software (Mathworks, Natic, MA) (37).

The posturographic assessment consisted of step-up and step-down tasks. The procedure was conducted as previously described (38). Each task comprised three repetitions, based on which the means of the study variables were calculated. Participants started all tasks with quiet standing and the feet positioned shoulder-width apart, arms alongside the trunk and eyes looking straight ahead. Platform change started at a sound signal. The step-up and step-down tasks were performed with the affected and unaffected limb in random order.

The recording of center of foot pressure (COP) displacements was divided into three phases: phase 1 - quiet standing before step-up/step-down, phase 2 - transit, and phase 3 - quiet standing until measurement completion. The recording was divided into phases using an algorithm that had already been presented by Stania et al. (38).

The following variables of COP displacement were determined:

1st phase and 3rd phase:

- sway range (raCOP) [cm] in the sagittal (_AP_) and frontal (_ML_) planes,
- mean velocity of COP (vCOP) [cm/s] in the sagittal (_AP_) and frontal (_ML_) planes.

2nd phase:

- transit time—time from exit from stability until gaining post-transit stability [s];
- double-support period—time when one foot was in contact with platform A and the other with platform B [s].

### Statistical analysis

The Shapiro–Wilk test was used to check the data for normal distribution while variance homogeneity was investigated using Levene’s test. The homogeneity of the patients’ age, body weight, standing height, duration of symptoms, intensity of activity-related pain and VISA-A score was analyzed with the Kruskal-Wallis test by ranks. The distribution of the remaining variables, i.e., sex, tendinopathy location (right vs. left limb), and BMI was tested using the Chi-square test of independence.

The three-way repeated measures ANOVA was used to analyze the posturographic parameters. A 3 × 2 × 2 (measurement time point × group × movement) and a 3 × 2 × 2 (measurement time point × group × limb condition) factorial designs were applied. All two and three-way interactions were analyzed. The ANOVA results were used to calculate the F-statistics for each main effect and interaction. The *post-hoc* comparisons were performed using the Bonferroni test. Mauchly’s sphericity test was used to validate a repeated measures ANOVA. The Greenhouse–Geisser correction was applied as a method of adjusting for lack of sphericity in a repeated measures ANOVA. The effect size was expressed as partial eta-squared. In all tests the level of statistical significance was set at *p* ≤ 0.05.

## Results

All three groups were homogeneous with respect to participant characteristics and baseline posturographic parameters recorded during the step-up and step-down tasks. However, groups A and B differed with respect to the number of female and male patients (31).

The three-way interactions between therapy type, limb condition, measurement time point and the type of the locomotor task were not significant for any of the measured variables. Although the three-way repeated measures ANOVA showed a few statistically significant two-factor interactions, the Bonferroni post-hoc test did not confirm statistical significance of the obtained results (Tables 2–4).

**Table 2:** Results of the three-way repeated measures ANOVA with a 3 × 2 × 2 factorial design (time point × group × movement) for radial shock wave and ultrasound therapy groups.

	Affected limb					Non-affected limb			
	Variable (unit)	Time point (T)	Group (G)	Movement (M)	Interactions	Time point (T)	Group (G)	Movement (M)	Interactions
Phase 1	raCOPap (cm)	NS	NS	NS	T*G *F*_(2,46)_ = 4.14 *p* = 0.022 *η*^2^ = 0.15	NS	NS	NS	NS
	raCOPml (cm)	NS	*F*_(1,23)_ = 5.88 *p* = 0.02 η^2^ = 0.2	NS	NS	*F*_(2,46)_ = 4.59 *p* = 0.015 *η*^2^ = 0.17	*F*_(1,23)_ = 5.13 *p* = 0.033 *η*^2^ = 0.18	NS	NS
	vCOPap (cm/s)	*F*_(2,46)_ = 15.23 *p* < 0.001 *η*^2^ = 0.4	*F*_(1,23)_ = 4.62 *p* = 0.04 *η*^2^ = 0.17	*F*_(1,23)_ = 4.48 *p* = 0.04 *η*^2^ = 0.16	NS	*F*_(2,46)_ = 13.247 *p* < 0.001 *η*^2^ = 0.37	*F*_(1,23)_ = 7.1 *p* = 0.014 *η*^2^ = 0.24	NS	T*M *F*_(2,46)_ = 3.91 *p* = 0.027 *η*^2^ = 0.15
	vCOPml (cm/s)	*F*_(2,46)_ = 8.12 *p* = 0.001 *η*^2^ = 0.26	*F*_(1,23)_ = 6.06 *p* = 0.02 *η*^2^ = 0.21	NS	NS	*F*_(2,46)_ = 10.53 *p* < 0.001 *η*^2^ = 0.31	*F*_(1,23)_ = 6.86 *p* = 0.015 *η*^2^ = 0.23	NS	NS
Phase 2	Transit time (s)	*F*_(2,46)_ = 7.47 *p* = 0.002 η^2^ = 0.25	NS	NS	NS	NS	NS	NS	NS
	Double-support period (s)	*F*_(2,46)_ = 32.83 *p* < 0.001 *η*^2^ = 0.59	NS	*F*_(1,23)_ = 5.85 *p* = 0.024 *η*^2^ = 0.2	NS	*F*_(2,46)_ = 42.84 *p* < 0.001 *η*^2^ = 0.65	NS	*F*_(1,23)_ = 13.18 *p* = 0.001 *η*^2^ = 0.36	T*M *F*_(2,46)_ = 6.58 *p* = 0.003 *η*^2^ = 0.22
Phase 3	raCOPap (cm)	NS	NS	NS	T*G *F*_(2,46)_ = 3.56 *p* = 0.036 *η*^2^ = 0.13	NS	NS	NS	T*G *F*_(2,46)_ = 4.09 *p* = 0.023 *η*^2^ = 0.15
	raCOPml (cm)	NS	NS	NS	NS	NS	NS	*F*_(1,23)_ = 6.4 *p* = 0.02 *η*^2^ = 0.22	M*G *F*_(1,23)_ = 4.3 *p* = 0.049 *η*^2^ = 0.16
	vCOPap (cm/s)	*F*_(2,46)_ = 6.1 *p* = 0.004 *η*^2^ = 0.21	NS	NS	NS	*F*_(2,46)_ = 9.64 *p* < 0.001 *η*^2^ = 0.3	NS	*F*_(1,23)_ = 4.52 *p* = 0.04 *η*^2^ = 0.16	NS
	vCOPml (cm/s)	NS	NS	NS	T*G *F*_(2,46)_ = 5.36 *p* = 0.008 *η*^2^ = 0.19	NS	NS	*F*_(1,23)_ = 6.15 *p* = 0.02 *η*^2^ = 0.21	M*G *F*_(1,23)_ = 7.14 *p* = 0.014 *η*^2^ = 0.24

NS, not significant; *η*^2^, effect size explained with partial eta-square.

**Table 3:** Results of the three-way repeated measures ANOVA with a 3 × 2 × 2 factorial design (time point × group × limb condition) for radial shock wave and ultrasound therapy groups.

		Step up				Step down			
	Variable (unit)	Time point (T)	Group (G)	Limb (L)	Interactions	Time point (T)	Group (G)	Limb (L)	Interactions
Phase 1	raCOPap (cm)	NS	NS	NS	NS	NS	NS	NS	NS
	raCOPml (cm)	NS	NS	NS	NS	*F*_(2,46)_ = 7.56 *p* = 0.001 *η*^2^ = 0.25	*F*_(1,23)_ = 8.29 *p* = 0.008 *η*^2^ = 0.27	NS	NS
	vCOPap (cm/s)	NS	NS	NS	NS	NS	*F*_(1,23)_ = 7.83 *p* = 0.01 *η*^2^ = 0.25	NS	NS
	vCOPml (cm/s)	NS	*F*_(1,23)_ = 5.83 *p* = 0.024 *η*^2^ = 0.202	NS	NS	NS	*F*_(1,23)_ = 7.04 *p* = 0.014 *η*^2^ = 0.23	NS	NS
Phase 2	Transit time (s)	NS	NS	NS	NS	NS	NS	NS	T*L *F*_(2,46)_ = 3.69 *p* = 0.033 *η*^2^ = 0.14
	Double-support period (s)	NS	NS	NS	T*L *F*_(1.56,35.92)_ = 4.81 *p* = 0.021 *η*^2^ = 0.17	*F*_(2,46)_ = 3.95 *p* = 0.026 *η*^2^ = 0.15	NS	NS	NS
Phase 3	raCOPap (cm)	NS	NS	NS	T*G *F*_(2,46)_ = 5.35 *p* = 0.008 *η*^2^ = 0.19	NS	NS	NS	NS
	raCOPml (cm)	NS	NS	NS	NS	*F*_(2,46)_ = 4.551 *p* = 0.016 *η*^2^ = 0.165	*F*_(1,23)_ = 4.319 *p* = 0.049 *η*^2^ = 0.158	NS	G*L *F*_(1,23)_ = 4.71 *p* = 0.041 *η*^2^ = 0.17
	vCOPap (cm/s)	NS	NS	NS	T*G *F*_(1.61,37.08)_ = 4.29 *p* = 0.028 *η*^2^ = 0.16	NS	NS	NS	NS
	vCOPml (cm/s)	NS	NS	NS	NS	NS	NS	NS	NS

NS, not significant; *η*^2^, effect size explained with partial eta-square.

**Table 4:** Results of the three-way repeated measures ANOVA with a 3 × 2 × 2 factorial design (time point × group × movement) for ultrasound therapy and ultrasound placebo groups.

		Affected limb				Non-affected limb			
	Variable (unit)	Time point (T)	Group (G)	Movement (M)	Interactions	Time point (T)	Group (G)	Movement (M)	Interactions
Phase 1	raCOPap (cm)	*F*_(2,46)_ = 11.06 *p* < 0.001 *η*^2^ = 0.33	NS	NS	NS	F_(2,46)_ = 8.47 *p* < 0.001 *η*^2^ = 0.27	NS	NS	NS
	raCOPml (cm)	*F*_(2,46)_ = 3.64 *p* = 0.034 *η*^2^ = 0.14	NS	NS	NS	*F*_(2,46)_ = 4.24 *p* = 0.02 *η*^2^ = 0.16	NS	NS	NS
	vCOPap (cm/s)	*F*_(2,46)_ = 27.34 *p* < 0.001 *η*^2^ = 0.54	NS	*F*_(1,23)_ = 11.18 *p* = 0.003 *η*^2^ = 0.33	T*M *F*_(2,46)_ = 4.39 *p* = 0.018 *η*^2^ = 0.16	*F*_(2,46)_ = 22.79 *p* < 0.001 *η*^2^ = 0.5	NS	*F*_(1,23)_ = 14.85 *p* < 0.001 *η*^2^ = 0.39	T*M *F*_(2,46)_ = 7.62 *p* = 0.001 *η*^2^ = 0.25
	vCOPml (cm/s)	*F*_(2,46)_ = 5.75 *p* = 0.006 *η*^2^ = 0.20	NS	NS	NS	*F*_(2,46)_ = 13.04 *p* < 0.001 *η*^2^ = 0.36	NS	NS	NS
Phase 2	Transit time (s)	*F*_(2,46)_ = 6.01 *p* = 0.005 *η*^2^ = 0.201	NS	NS	NS	NS	NS	NS	NS
	Double-support period (s)	*F*_(2,46)_ = 45.32 *p* < 0.001 *η*^2^ = 0.66	NS	*F*_(1,23)_ = 10.02 *p* = 0.004 *η*^2^ = 0.30	NS	*F*_(2,46)_ = 49.02 *p* < 0.001 *η*^2^ = 0.68	NS	*F*_(1,23)_ = 33.2 *p* < 0.001 *η*^2^ = 0.59	T*M *F*_(2,46)_ = 5.96 *p* = 0.005 *η*^2^ = 0.21
Phase 3	raCOPap (cm)	*F*_(2,46)_ = 5.13 *p* = 0.01 *η*^2^ = 0.18	NS	NS	NS	NS	NS	NS	T*G *F*_(2,46)_ = 3.83 *p* = 0.029 *η*^2^ = 0.14
	raCOPml (cm)	*F*_(2,46)_ = 6.16 *p* < 0.004 *η*^2^ = 0.21	NS	NS	NS	NS	NS	NS	T*G *F*_(2,46)_ = 4.57 *p* = 0.015 *η*^2^ = 0.17 T*M *F*_(2,46)_ = 4.92 *p* = 0.011 *η*^2^ = 0.17
	vCOPap (cm/s)	*F*_(2,46)_ = 3.71 *p* < 0.032 *η*^2^ = 0.14	NS	NS	NS	*F*_(2,46)_ = 12.02 *p* < 0.001 *η*^2^ = 0.34	NS	NS	NS
	vCOPml (cm/s)	NS	NS	NS	NS	NS	NS	NS	NS

NS, not significant; *η*^2^, effect size explained with partial eta-square.

### The effect of therapy type (radial shock wave therapy vs. ultrasound therapy), time point of measurement, and type of the locomotor task on the posturographic parameters for the affected and non-affected limbs

**Figure 2:**
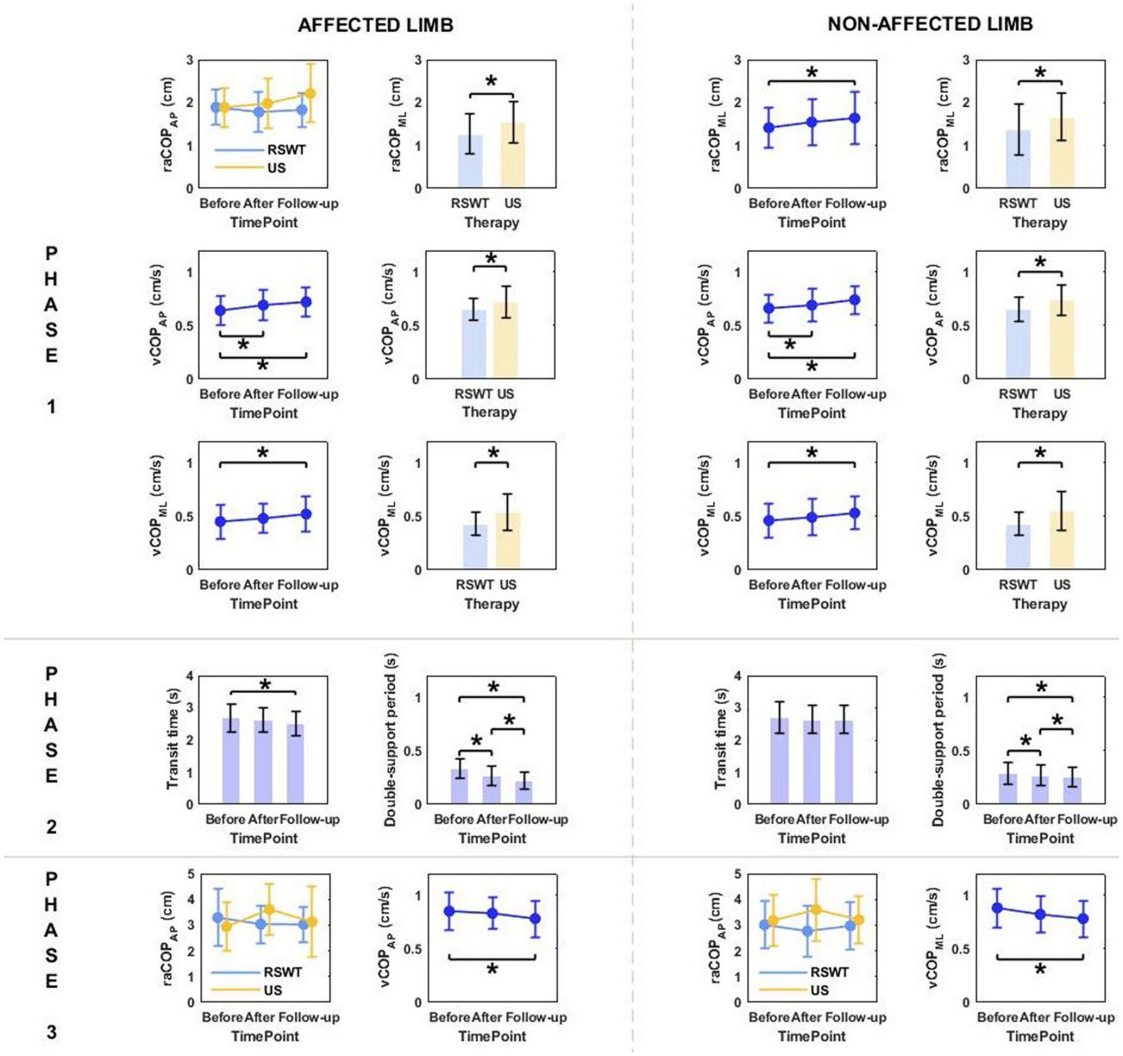
The significant differences in posturographic parameters for affected and non-affected limbs of patients from RSWT (radial shock wave therapy) and US (ultrasound) groups in relation to time point and therapy type. *indicates statistically significant differences.

Phase 1: The three-way repeated measures ANOVA with a 3 × 2 × 2 factorial design (measurement time point × group × movement) revealed a group effect on raCOP_ML_, vCOP_AP_, and vCOP_ML_ of the transit initiated by the affected and non-affected limbs (Table 2). The Bonferroni post-hoc test showed the means of all those variables were significantly smaller for the RSWT group than ultrasound group (*p* < 0.05) (Figure 2). Also, measurement time point had an effect on the vCOP_AP_, vCOP_ML_ of the transit initiated by the affected limb and raCOP_ML_, vCOP_AP_, and vCOP_ML_ of the transit initiated by the non-affected limb (Table 2). The *post-hoc* test confirmed significantly greater values of those variables at 6 weeks after therapy compared to baseline (Figure 2).

**Figure 3:**
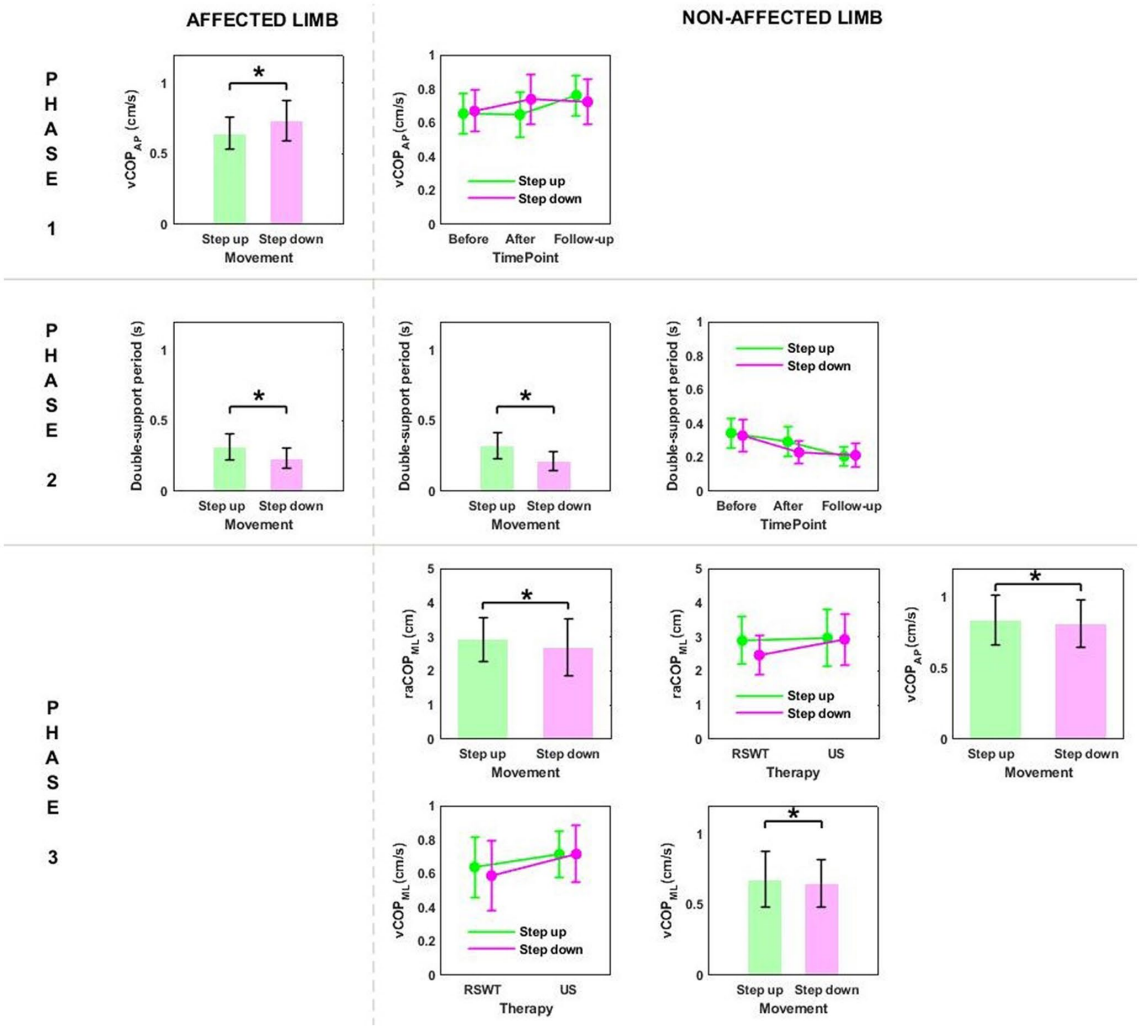
The significant differences in posturographic parameters for affected and non-affected limbs of patients from RSWT (radial shock wave therapy) and US (ultrasound) groups in relation to the type of locomotor task. *indicates statistically significant differences.

Phase 2: The three-way ANOVA showed measurement time point had an effect on most of the variables measured in phase 2, i.e., the time of transit initiated by the affected limb and the legnth of double-support period for transit initiated by the affected and non-affected limbs (Table 2). All patients who underwent RSWT and real ultrasound interventions presented significantly shorter transit time and double-support period at 6 weeks after therapy completion compared to baseline (Figure 2). The type of the locomotor task was also found to have a significant effect on the length of double support period for transit initiated by the affected and non-affected limbs. The post-hoc test showed double-support was shorter for the step-down task compared to step-up (Figure 3).

Phase 3: The three-way ANOVA demonstated a significant effect of the locomotor task type on raCOP_ML_, vCOP_AP_, and vCOP_ML_ of the transit initiated by the non-affected limb (Table 2). The variables were significantly lower for the step-down task compared to step-up (*p* < 0.05) (Figure 3).

### The effect of therapy type (radial shock wave therapy vs. ultrasound therapy), time point of measurement, and limb condition on the posturographic parameters of the step-up and step-down tasks

**Figure 4:**
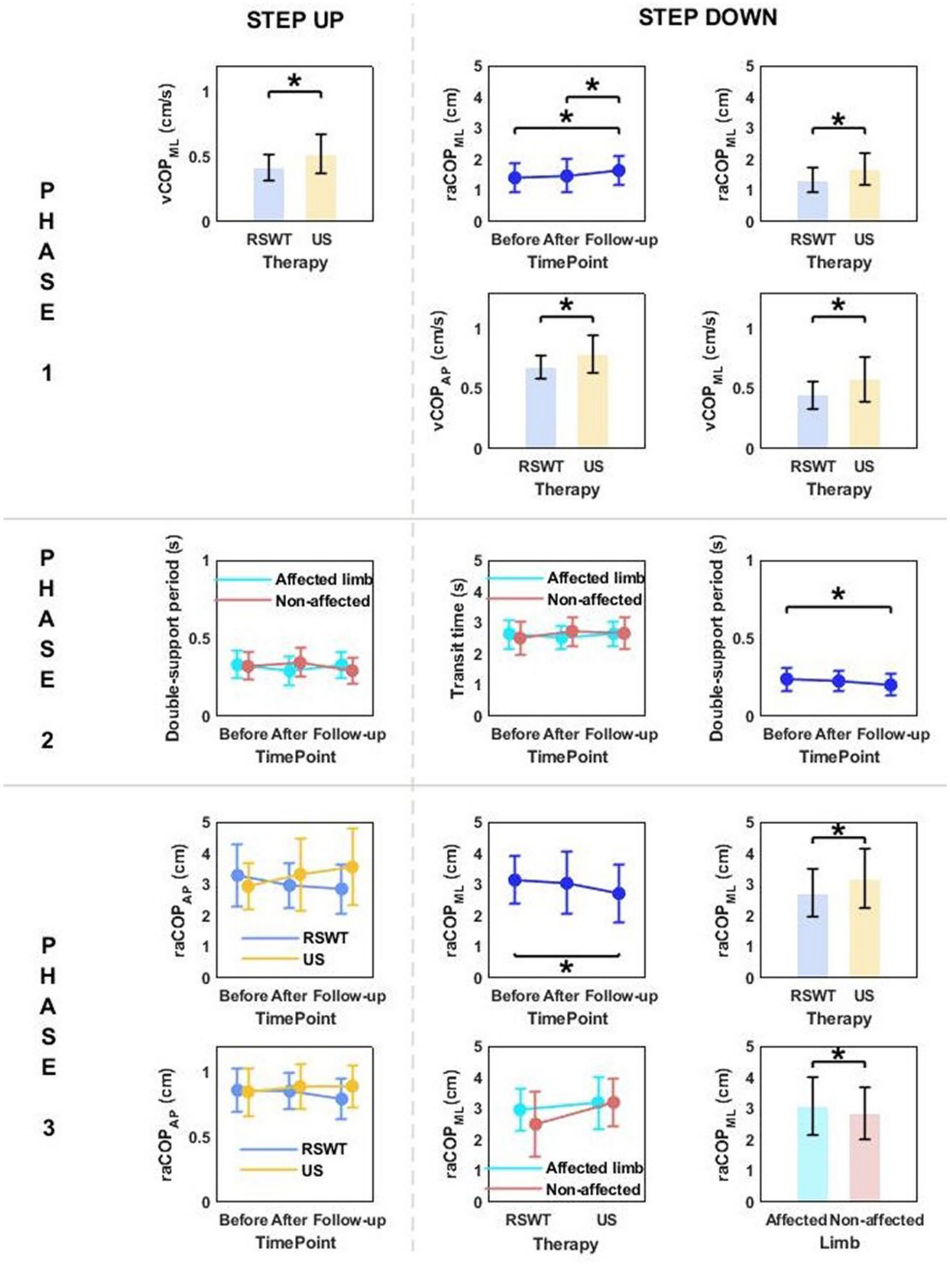
The significant differences in posturographic parameters for step-up and step-down tasks in patients from RSWT (radial shock wave therapy) and US (ultrasound) groups in relation to time point and therapy type. *indicates statistically significant differences.

Phase 1: The three-way repeated measures ANOVA with a 3 × 2 × 2 factorial design (measurement time point × group × limb condition) revealed a group effect on vCOP_ML_ for the step-up task, and raCOP_ML_, vCOP_AP_, vCOP_ML_ for the step-down task (Table 3). The Bonferroni post-hoc test showed that the means of all variables were significantly smaller for the RSWT group than ultrasound group (*p* < 0.05) (Figure 4).

Phase 3: A significant effect of therapy type on raCOP_ML_ for the step-down task was also observed (Table 3). The ultrasound group had significantly larger sway range in the frontal plane than the RSWT group (*p* < 0.05) (Figure 4).

### The effect of therapy type (ultrasound vs. placebo ultrasound), time point of measurement, and the type of the locomotor task on the posturographic parameters for affected and non-affected limbs

**Figure 5:**
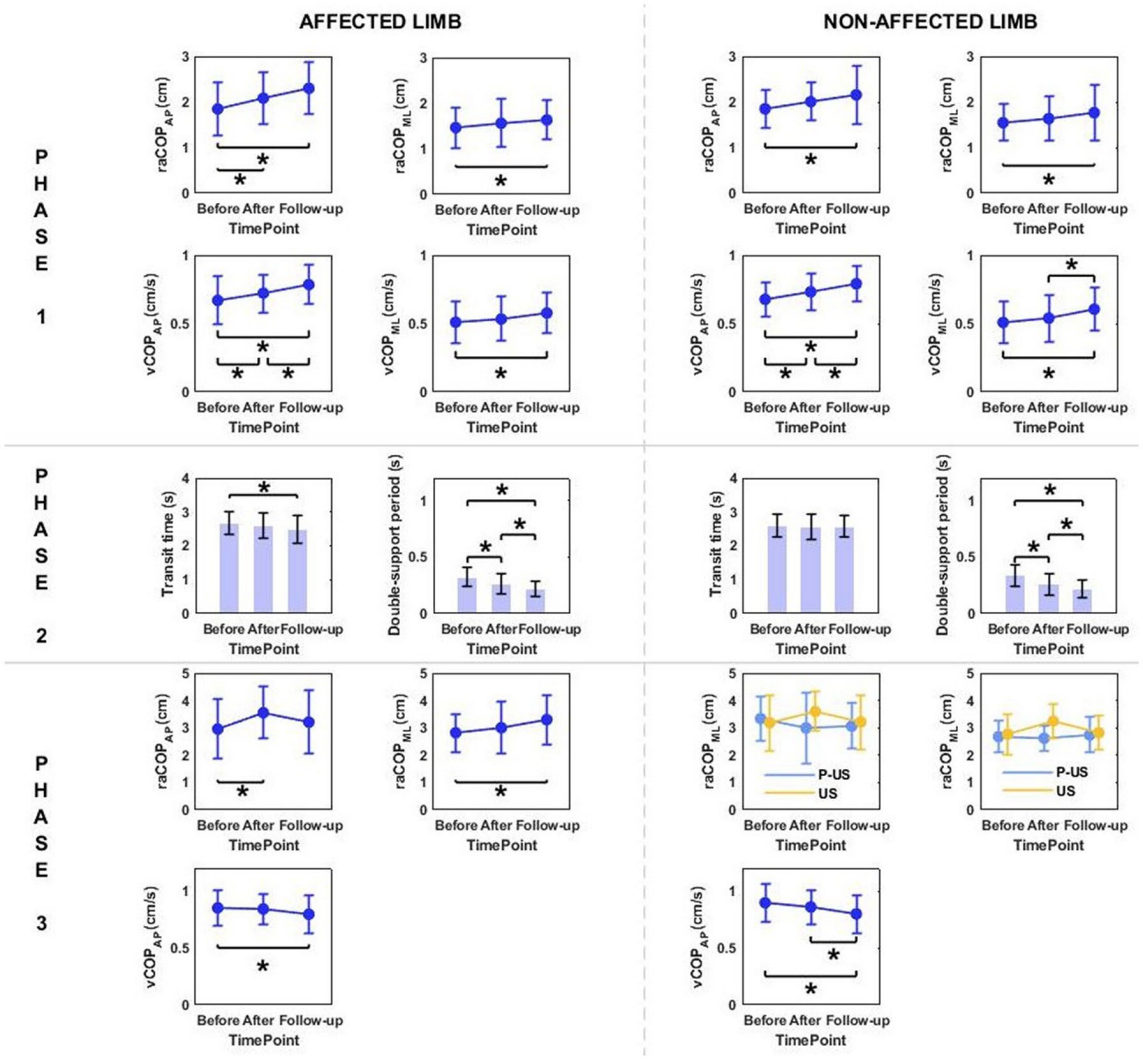
The significant differences in posturographic parameters for affected and non-affected limbs of patients from US (ultrasound) and placebo US groups in relation to time point. *indicates statistically significant differences.

**Figure 6:**
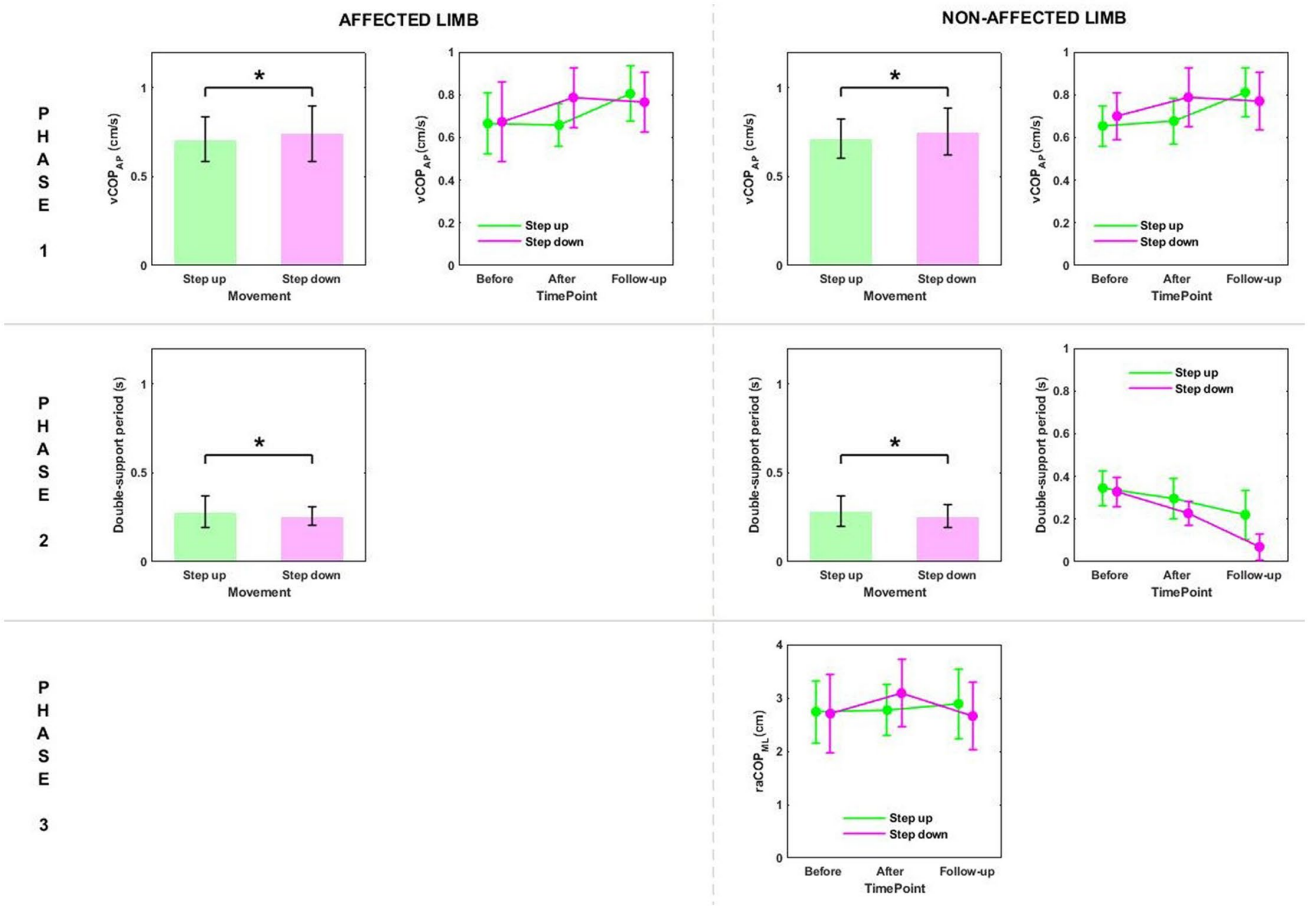
The significant differences in posturographic parameters for affected and non-affected limbs of patients from US (ultrasound) and placebo US groups in relation to the type of locomotor task. *indicates statistically significant differences.

Phase 1: The three-way repeated measures ANOVA with a 3 × 2 × 2 factorial design (time point × group × movement) revealed a time point effect on all analyzed variables (Table 4). The Bonferroni *post-hoc* test showed all variables exhibited a gradual increase. Compared to baseline, the highest values were reached at 6 weeks of therapy completion; the difference was statistically significant (*p* < 0.05) (Figure 5). The three-way ANOVA also revealed a significant effect of the locomotor task type (initiated by the affected and non-affected limbs) on vCOP_AP_ (Table 4). Step-down vCOP_AP_ was significantly lower compared to the step-up task (*p* < 0.05) (Figure 6).

Phase 2: Measurement time point had an effect on the time of transit initiated by the affected limb and the legnth of double-support period for transit initiated by the affected and non-affected limbs (Table 4). At 6 weeks post-therapy, transit time and double-support period were significantly shorter compared to baseline (*p* < 0.05) (Figure 5). The three-way ANOVA also revealed a significant effect of the locomotor task type on the legnth of double-support period for transit initiated by the affected and non-affected limbs (Table 4), which was significantly longer for the step-up task (*p* < 0.05) (Figure 6).

### The effect of therapy type (ultrasound vs. placebo ultrasound), time point of measurement, and limb condition on the posturographic parameters of the step-up and step-down tasks

**Figure 7:**
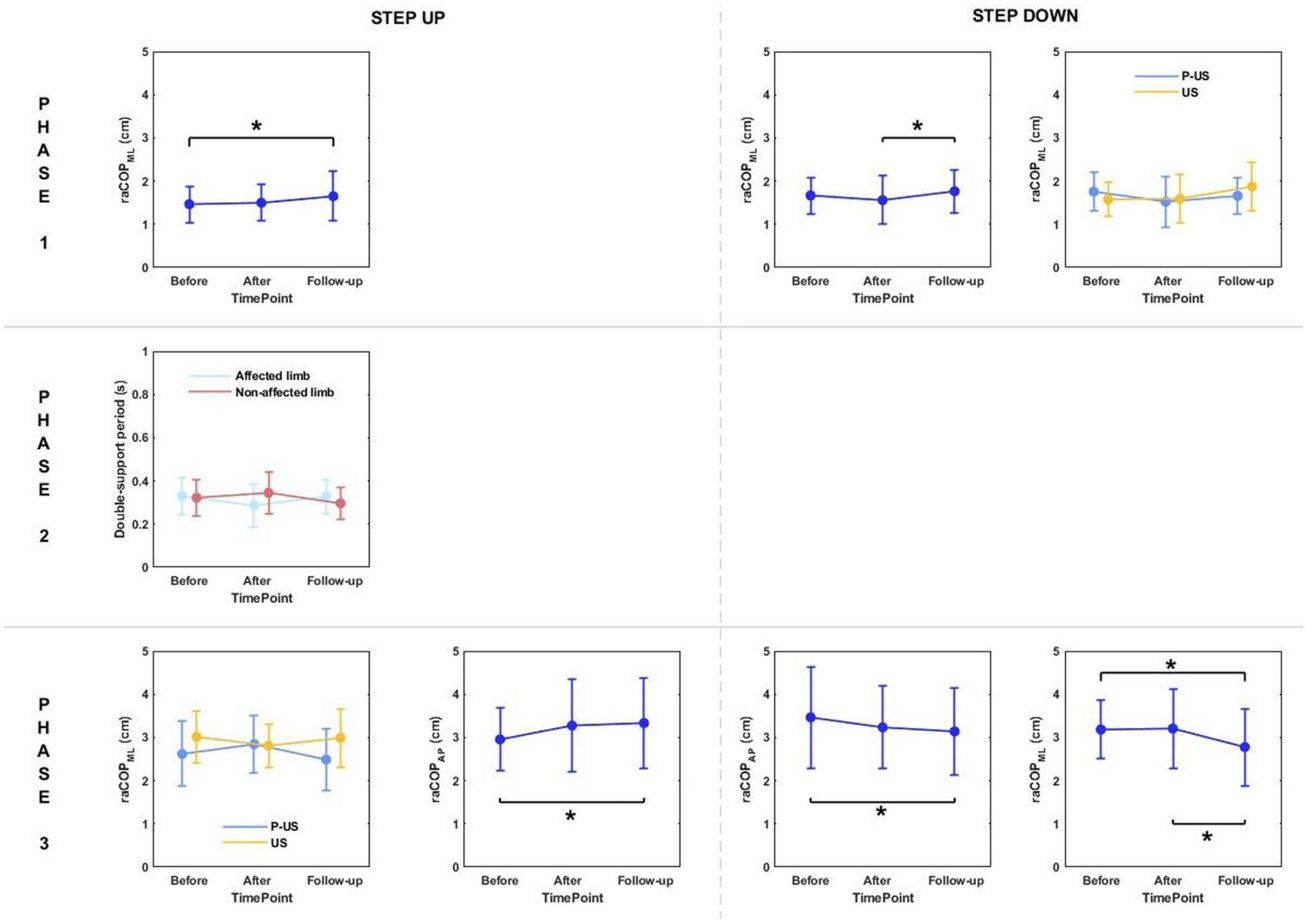
The significant differences in posturographic parameters for step-up and step-down tasks in patients from US (ultrasound) and placebo US groups in relation to time point and therapy type. *indicates statistically significant differences.

Phase 1: The three-way repeated measures ANOVA with a 3 × 2 × 2 factorial design (measurement time point × group × limb condition) revealed a time point effect on raCOP_ML_ for the step-up and step-down tasks (Table 5). Sway range in the frontal plane was significantly larger at 6 weeks post-therapy compared to baseline (for the step-up task) and 1 week post-therapy (for the step-down task) (*p* < 0.05) (Figure 7).

**Table 5:** Results of the three-way repeated measures ANOVA with a 3 × 2 × 2 factorial design (time point × group × limb condition) for ultrasound therapy and ultrasound placebo groups.

		Step up				Step down			
	Variable (unit)	Time point (T)	Group (G)	Limb (L)	Interactions	Time point (T)	Group (G)	Limb (L)	Interactions
Phase 1	raCOPap (cm)	NS	NS	NS	NS	NS	NS	NS	NS
	raCOPml (cm)	*F*_(2,46)_ = 4.959 *p* = 0.011 *η*^2^ = 0.177	NS	NS	NS	*F*_(2,46)_ = 4.705 *p* = 0.014 *η*^2^ = 0.17	NS	NS	T*G *F*_(2,46)_ = 4.35 *p* = 0.019 *η*^2^ = 0.16
	vCOPap (cm/s)	NS	NS	NS	NS	NS	NS	NS	NS
	vCOPml (cm/s)	NS	NS	NS	NS	NS	NS	NS	NS
Phase 2	Transit time (s)	NS	NS	NS	NS	NS	NS	NS	NS
	Double-support period (s)	NS	NS	NS	T*L *F*_(2,46)_ = 8.22 *p* < 0.001 *η*^2^ = 0.26	NS	NS	NS	NS
Phase 3	raCOPap (cm)	*F*_(2,46)_ = 3.539 *p* = 0.037 *η*^2^ = 0.133	NS	NS	NS	*F*_(2,46)_ = 3.652 *p* = 0.034 *η*^2^ = 0.137	NS	NS	NS
	raCOPml (cm)	NS	NS	NS	T*G *F*_(2,46)_ = 3.67 *p* = 0.033 *η*^2^ = 0.14	*F*_(2,46)_ = 6.292 *p* = 0.004 *η*^2^ = 0.215	NS	NS	NS
	vCOPap (cm/s)	NS	NS	NS	NS	NS	NS	NS	NS
	vCOPml (cm/s)	NS	NS	NS	NS	NS	NS	NS	NS

NS, not significant; *η*^2^, effect size explained with partial eta-square.

Phase 3: The three-way ANOVA revealed a significant effect of measurement time point on raCOP_AP_ for the step-up task (Table 5). The values obtained at 6 weeks of therapy completion were significantly greater compared to baseline (Figure 7).

## Discussion

The three-way ANOVA did not confirm superiority of any of the three therapeutic interventions used in patients with non-insertional Achilles tendinopathy indicating dynamic posturography testing on force platforms during step ascent/descent does not provide reliable information on the mechanotherapy-related healing of the affected Achilles tendon. Nevertheless, irrespective of therapy type and locomotor task, a significant increase in the postural sway was observed in the entire study population and throughout the follow-up period, which seems to partly support our research hypothesis.

In a laboratory setting, gait is assessed using 3D-Gait Analysis system consisting of an optoelectronic system, dynamographic platforms and video cameras (26, 29, 39). In clinical practice (physiotherapist-patient), such complex diagnostic systems are rarely used due to high costs, lack of access to a sufficiently large measurement room and the lengthy and complicated analysis of results (40). In our experiment, two force platforms were used that allowed for posturographic assessment of step-up and step-down initiation, i.e., common everyday activities.

Step initiation represents a transitional period between quiet standing and gait. It is a functional act that is used in research as a classical paradigm to study postural control during movement when changes occur in base of support and in the position of the body’s center of gravity (41). Feed-forward postural adjustments are an important feature of the postural control system (42) that involves two mechanisms, i.e., anticipatory postural adjustments (APAs) and early postural adjustements (EPAs) (43). EPAs are aimed at minimizing the mechanical effects of a planned action and/or expected balance perturbation which may occur in the case of step-up and step-down tasks. APAs, on the other hand, generate net forces and moments of force that counteract movement-related perturbation (43). None of the patients in this experiment had trouble regaining stability after the step-up or step-down trial, which demonstrated efficient EPAs and correct active control of the antigravity muscles prior to foot contact (44).

The human standing posture is described as an inverted pendulum model rotating about the ankle joint. The reduction of postural sway is also associated with increased stiffness in this joint due to muscle tension, mainly affecting the anti-gravity muscles that stabilise this joint (45). The characteristic clinical features of Achilles tendinopathy are pain, swelling and stiffness after prolonged periods of rest. As a consequence, patients experience increased tension of the gastrocnemius muscle. A recently published analysis of the results obtained in the first part of our research project (31) revealed the parameters of COP trajectories in the sagittal plane were significantly greater for the non-affected limb compared to the limb with Achilles tendinopathy. In the present study, the repeated measures ANOVA revealed a time point effect on most of the measured variables. Successive measurements showed that raCOP and vCOP exhibited a gradual and statistically significant increase, while the temporal parameters (transit time and double-support period) decreased significantly irrespective of therapy type and locomotor task. It should be noted though that all patients received deep friction massage as the primary therapy (46) observed that soft tissue mobilization in a rabbit model of Achilles tendinopathy promoted collagen fibre realignment, increased cross-sectional area and improved viscoelasticity of the treated tendons. It can therefore be speculated that the significantly lower raCOP and vCOP values in the sagittal and frontal planes recorded before the start of the experiment might have resulted from a change in the viscoelastic properties of the ankle’s musculotendinous units. Such changes are characteristic of collagen fiber breakdown. The gradual increase in postural sway over the observation period was probably related to subsidence of tendinopathy symptoms (as confirmed by (31)) and relief of ankle soft tissue tension due to therapies combined with deep friction massage.

Stair ascent turned out to be a more demanding biomechanical task compared to stair descent for young healthy subjects (29). Stair dimensions and the mean body height seemed to influence the temporal and angular kinematics of the lower limb during stairclimbing (47). In our experiment, the groups were homogeneous in terms of body height. As predicted, the three-way ANOVA showed a statistically significant effect of the type of the locomotor task on the double-support time for both limbs, which was significantly longer during step-up than during step-down (Figures 3, 6).

In accordance with the research hypothesis, the three-way ANOVAs used for intergroup comparison (radial shock wave vs. ultrasound) revealed a group effect on almost all measured variables in phase 1, i.e., quiet standing before step-up/step-down (Figure 2). Overall, postural stability before the step-up and step-down trials turned out to be more efficient in patients who received RSWT compared to the ultrasound group, irrespective of the locomotor task type, lower limb condition and time elapsed since therapy. It can be speculated that intergroup differences in postural sway resulted from a markedly greater mechanical impact of the shock waves on the gastrocnemius-Achilles tendon complex compared to the effect of ultrasound waves. The inverted pendulum model of upright standing is mostly based on the contribution of ankle plantarflexors/dorsiflexors to quiet standing in the sagittal plane (45). The results of Shin et al. (48) implied that upright postural control was partly related to intrinsic muscle stiffness in the lower limbs. It is likely that the shock wave changed the contractile properties of the muscle-tendon unit of the ankle joint to a greater extent than ultrasound and therefore had a greater effect on postural control Shock waves are capable of inducing cellular and molecular changes that promote the regeneration of damaged tissues (49).

Tendon regeneration processes that occur after shock wave application have also been accounted for by the stimulation of tenocyte proliferation and collagen synthesis (50, 51). Ultrasound, like a shock wave, is a form of mechanical energy that can alter collagen content and alignment, and stimulate tendon cell migration and proliferation (52, 53); however, the impact of ultrasound stimulation is markedly smaller.

The main limitation of this study was a small number of patients in the experimental groups. The sample size was calculated based on the means and standard deviations of the subjective outcomes of the pilot study (pain intensity and VISA-A score) (31). As our unpublished study did not include a posturographic test, no effect size was calculated for posturographic parameters. Another limitation was lack of long-term follow-up. Although the research design predicted a long-term measurement at 6 months after the completion of therapeutic interventions, only 60% of the patients attended the last measurement session. According to ICON group recommendations (35), when planning a subsequent randomized controlled trial, we will try to include a measure for each of the nine core domains for tendinopathy at a minimum, so that future meta-analyses might be able to better estimate therapeutic effects.

## Conclusion

Objective posturographic assessment during step-up and step-down initiation did not demonstrate therapeutic superiority of any of the three therapeutic interventions used in patients with non-insertional Achilles tendinopathy. Regardless of the therapy used, all patients showed significant increases in postural sway throughout the follow-up period. In addition, irrespective of the time point of measurement and the complexity of the locomotor task, the patients of the RSWT group had less difficulty controlling their postural balance before step initiation compared to those who received ultrasound therapy.

## Data availability statement

The original contributions presented in the study are included in the article/Supplementary material, further inquiries can be directed to the corresponding author.

## Ethics statement

The studies involving human participants were reviewed and approved by The Research Ethics Committee from The Academy of Physical Education in Katowice. The patients/participants provided their written informed consent to participate in this study.

## Author contributions

MS: conceptualization, methodology, investigation, writing—original draft preparation, and supervision. MP: formal analysis, review and editing of the manuscript. WM: investigation, software, review and editing of the manuscript. GJ: methodology, review and editing of the manuscript. KS: methodology, review and editing of the manuscript. PK: conceptualization, methodology, investigation, review and editing of the manuscript. All authors contributed to the article and approved the submitted version.

## Funding

This work was supported by the Academy of Physical Education in Katowice, Poland.

## Conflict of interest

The authors declare that the research was conducted in the absence of any commercial or financial relationships that could be construed as a potential conflict of interest.

## Publisher’s note

All claims expressed in this article are solely those of the authors and do not necessarily represent those of their affiliated organizations, or those of the publisher, the editors and the reviewers. Any product that may be evaluated in this article, or claim that may be made by its manufacturer, is not guaranteed or endorsed by the publisher.
